# Exploring the functional role of the CHRM2 gene in human cognition: results from a dense genotyping and brain expression study

**DOI:** 10.1186/1471-2350-8-66

**Published:** 2007-11-08

**Authors:** Florencia M Gosso, Eco JC de Geus, Tinca JC Polderman, Dorret I Boomsma, Danielle Posthuma, Peter Heutink

**Affiliations:** 1 Dept of Biological Psychology, Vrije Universiteit, Amsterdam, The Netherlands; 2 Section of Medical Genomics, Department of Clinical Genetics, VU Medical Center, Amsterdam, The Netherlands; 3 Center for Neurogenomics and Cognitive Research – CNCR, Vrije Universiteit, Amsterdam, The Netherlands

## Abstract

**Background:**

The *CHRM2 *gene, located on the long arm of chromosome 7 (7q31-35), is involved in neuronal excitability, synaptic plasticity and feedback regulation of acetylcholine release, and has been implicated in higher cognitive processing. The aim of this study is the identification of functional (non)coding variants underlying cognitive phenotypic variation.

**Methods:**

We previously reported an association between polymorphisms in the 5'UTR regions of the *CHRM2 *gene and intelligence.. However, no functional variants within this area have currently been identified. In order to identify the relevant functional variant(s), we conducted a denser coverage of SNPs, using two independent Dutch cohorts, consisting of a children's sample (N = 371 ss; mean age 12.4) and an adult sample (N= 391 ss; mean age 37.6). For all individuals standardized intelligence measures were available. Subsequently, we investigated genotype-dependent *CHRM2 *gene expression levels in the brain, to explore putative enhancer/inhibition activity exerted by variants within the muscarinic acetylcholinergic receptor.

**Results:**

Using a test of within-family association two of the previously reported variants – rs2061174, and rs324650 – were again strongly associated with intelligence (*P *< 0.01). A new SNP (rs2350780) showed a trend towards significance. SNP rs324650, is located within a short interspersed repeat (SINE). Although the function of short interspersed repeats remains contentious, recent research revealed potential functionality of SINE repeats in a gene-regulatory context. Gene-expression levels in post-mortem brain material, however were not dependent on rs324650 genotype.

**Conclusion:**

Using a denser coverage of SNPs in the *CHRM2 *gene, we confirmed the 5'UTR regions to be most interesting in the context of intelligence, and ruled out other regions of this gene. Although no correlation between genomic variants and gene expression was found, it would be interesting to examine allele-specific effects on CHRM2 transcripts expression in much more detail, for example in relation to transcripts specific halve-life and their relation to LTP and memory.

## Background

Identifying genes for variation in the range of normal intelligence could provide important clues to the genetic etiology of disturbed cognition in e.g. autism, reading disorder, and ADHD. Since the earliest 90's several groups have focussed on the identification – and subsequent replication – of common genetic polymorphisms underlying normal variation in cognitive abilities [1-5]. Among a handful of candidate genes that have been investigated in relation to normal cognitive variation as summarized in Posthuma & De Geus 2006 [6], the muscarinic 2 cholinergic receptor gene (*CHRM2*) has been consistently found to be associated with cognitive ability, and currently is the best replicated gene associated with general intelligence. A population-based association study conducted by Comings *et al*. (2003) [7] reported an association between a 3'UTR variant of the cholinergic muscarinic receptor 2 (*CHRM2*) gene explaining 1% of the variance in scores on full-scale IQ (FSIQ), and years of education. Suggestive evidence for linkage with performance IQ was found at 7q31-36, in the vicinity of the *CHRM2 *gene in a genome scan for intelligence based on 329 Australian families and 100 Dutch families, totalling 625 sib-pairs [4]. We subsequently reported association between genetic variants within the *CHRM2 *gene and intelligence quotient (IQ) using two independent Dutch cohorts [8]. This finding was then replicated by Dick and colleagues [9]. All three association studies (Comings *et al*., 2003; Gosso *et al*., 2006; Dick *et al*., 2007) report significant association with IQ and non coding regions within in the *CHRM2 *gene (rs81919992 located in the 3' untranslated region (UTR) [7], and rs2061174 [9], and rs324650 [8] in introns 4 and 5, respectively).

The *CHRM2 *gene belongs to the superfamily of G-protein-coupled receptors (GPCRs). Muscarinic acetylcholine receptors (M_1_-M_5_) activate a multitude of signaling pathways important for modulating neuronal excitability, synaptic plasticity and feedback regulation of acetylcholine (ACh) release [10,11]. Combined behavioral and pharmacological animal studies involving M_2 _antagonists have shown the importance of cholinergic receptor activity for acquisition and retrieval of several learning tasks [12-16].

Despite its confirmed putative role in cognitive processes, further evidence for genetic regulatory variants on the *CHRM2 *gene have been difficult to assess, mainly due to its complex transcriptional expression patterns. Three different *CHRM2 *promoters have been reported based on work performed on different human cell lines [17]. In combination with *alternative *splicing patterns this results in, at least, 6 different mRNA transcripts encoding for the same receptor protein (isoforms A till F)[17,18]. Promoter activity for the *CHRM2 *gene was postulated to be tissue specific. The first promoter located upstream of exon 1, was preferentially used in cardiac cells (isoforms A and B); promoter 2 on intron 1 alternatively expressed on brain (isoforms C and D); and a third promoter located on intro 2 non-tissue specific (isoforms E and F). Independently, Zhou and coworkers [19] reported a fourth putative promoter region on intron 5, but this last result has not been independently confirmed yet [17]. Although *CHRM2 *promoter usage is believed to be tissue specific, a single protein receptor is encoded. The functional significance of these transcripts is still unknown.

To fine-map the *CHRM2 *gene and to detect its functional role in cognitive ability, we genotyped a dense set of *tag*-SNPs within and flanking the *CHRM2 *gene in a sample of 762 Dutch individuals from 358 twin families belonging to two different age cohorts (mean ages 12.4 and 37.6). A family based genetic association test was used, which allows evaluating evidence for association free from spurious effects of population stratification [20-22]. In addition, gene expression assays were performed on brain controls to determine whether a significant correlation exists between the associated SNPs and CHRM2 gene expression levels.

## Methods

### Subjects

All young and adult twins and their siblings were part of two larger cognitive studies and were recruited from the Netherlands Twin Registry [23,24]. We have shown previously that the adult participants are representative of the Dutch population with respect to intelligence [25]. Informed consent was obtained from the participants (adult cohort) or from their parents if they were under 18 (young cohort). The study was approved by the institutional review board of the VU University Medical Center. None of the individuals tested suffered from severe physical or mental handicaps, as assessed through surveys sent out to participants or their parents every two years.

### Young Cohort

The young cohort consisted of 177 twin pairs born between 1990 and 1992, and 55 siblings [6,26], of which 371 were available for genotyping. Mean age of the genotyped twins was 12.4 (SD = 0.9) years of age and the siblings were between 8 and 15 years old at the time of testing. There were 35 monozygotic male twin pairs (MZM), 28 dizygotic male twin pairs (DZM), 48 monozygotic female twin pairs (MZF), 23 dizygotic female twin pairs (DZF), 26 dizygotic opposite-sex twin pairs (DOS), 24 male siblings and 24 female siblings, and 3 subjects form incomplete twin pairs (1 male, 2 females). Participation in this study included a voluntary agreement to provide buccal swabs for DNA extraction.

This sample is similar to the sample used in our initial analyses, except for twenty individuals that were deleted from analyses in the current sample due to additional genotyping and a more stringent threshold of genotyping failure per individual.

### Adult Cohort

A total of 793 family members from 317 extended twin families participated in the adult cognition study [4]. Participation in this study did not automatically include DNA collection, however, part of the sample (276 subjects) returned to the lab to provide blood samples. The sample characteristics have been described elsewhere [27]. One hundred fifteen additional individuals provided buccal swabs via our biobanking project [28] for DNA extraction. Mean age of the total genotyped sample was 36.2 years (SD = 12.6). There were 25 monozygotic male twin pairs (MZM), 15 dizygotic male twin pairs (DZM), 1 DZM triplet, 20 monozygotic female twin pairs (MZF), 28 dizygotic female twin pairs (DZF) and 23 dizygotic opposite-sex twin pairs (DOS), 29 female siblings and 28 male siblings, and 109 subjects from incomplete twin pairs (41 males, 68 females).

### Cognitive testing

In the young cohort, cognitive ability was assessed with the Dutch adaptation of the WISC-R [29], and consisted of four verbal subtests (similarities, vocabulary, arithmetic, and digit span) and two performance subtests (block design, and object assembly).

In the adult cohort, the Dutch adaptation of the WAISIII-R [30], assessed IQ and consisted of four verbal subtests (VIQ: information, similarities, vocabulary, and arithmetic) and four performance subtests (PIQ: picture completion, block design, matrix reasoning, and digit-symbol substitution). The correlation between verbal IQ and performance IQ is usually around 0.50 (0.53 in our data), implying that only 25% of the variance in PIQ and VIQ is shared. Thus, a substantial part of the variance in these two measures is non-overlapping, and theoretically they are expected to capture different aspects of cognitive ability. We therefore included VIQ and PIQ as measures of the two different aspects of intelligence as well as Full scale IQ (FSIQ) as a general measure of intelligence. In both cohorts, VIQ, PIQ and FSIQ were normally distributed, (see Table 1).

**Table 1:** Means and standard deviations of IQ (corrected for age and sex effects) in the Young and Adult cohorts

	*Young Cohort*				*Adult Cohort*			
	*Total sample*	*Skewness Kurtosis*	*Genotyped*	*Skewness Kurtosis*	*Total sample*	*Skewness Kurtosis*	*Genotyped*	*Skewness Kurtosis*

N	407		371		793		391	
Gender (M/F)	191/216		176/195		348/445		175/216	
Age (SD)	12.37 (0.93)		12.37 (0.92)		37.60 (13.00)		36.25 (12.64)	
PIQ (SD)	94.57 (18.93)	0.165/-0.308	94.85 (19.14)	0.175/-0.304	104.49 (12.34)	0.197/0.099	104.30 (11.64)	0.135/0.312
VIQ(SD)	102.56 (12.74)	0.121/0.242	102.64 (12.92)	-0.080/-0.332	103.69 (12.26)	-0.308/-0.005	104.23 (12.15)	-0.410/0.256
FSIQ (SD)	98.65 (15.06)	-0.042/-0.252	98.84 (15.24)	-0.037/-0.254	103.56 (11.49)	0.087/0.167	103.81 (11.16)	0.073/0.512

For both cohorts IQ scores standardized for the effects of age and sex were calculated. These were then z-transformed within cohorts to allow easy comparison across cohorts and across different tests.

### DNA collection and isolation

Buccal swabs were collected from 371 children; DNA in adults was collected from blood samples in 391 adults. The DNA isolation from buccal swabs was performed using a cloroform/isopropanol extraction [31,32]. DNA was extracted from blood samples using the salting out protocol described elsewhere [33]. Zygosity was assessed using 11 highly polymorphic microsatellite markers (Heterozygosity > 0.80). Genotyping was performed blind to familial status and phenotypic data.

### DNA and RNA extraction from tissue homogenates

Control brains from 50 individuals, 23 males with a mean age of 70.3 years (SD = 9.38), and 27 females with a mean age of 73.3 years (SD = 10.50) were obtained at autopsy from *The Netherlands Brain Bank *(NBB) [34]. This material comes mainly from the superior and inferior parietal lobe. DNA isolation from 0.20 gram of frozen tissue was performed using the Puregene™ Kit (Gentra Systems, USA) according to standard protocol and doubled volume of all reagents per tissue weight. To verify DNA isolation, products were run on a 1% agarose gel.

Total RNA was isolated from 0.10 gram of frozen brain tissue with RNA-Bee™ following the manufacturer's recommendations (Isotex Diagnostics, Inc., USA). RNA was purified using the Qiagen RNeasy Mini kit (Qiagen Benelux B.V., The Netherlands) and verified on a 2% agarose gel. Five μg RNA was used to make cDNA using 200 U of Superscript™ III Reverse Transcriptase (Invitrogen, The Netherlands) in First Strand Buffer (Invitrogen, The Netherlands), 3.4 * 10^-2 ^μg/μl random hexamer oligo, 3.4 * 10^-2 ^μg/μl poly d(T) 12–18, 1.3 mM dNTPs, 1.1 μM DTT (Invitrogen, The Netherlands), 10 U RNaseOUT™ Ribonuclease Inhibitor Recombinant (Invitrogen, The Netherlands) and incubated two hours at 50°C. Subsequently, 20 U RNase H (Invitrogen, The Netherlands) was added and incubated 30 minutes at 37°C. Products were run on a 1% agarose gel to examine the quality.

### Genotyping

Single nucleotide polymorphisms (SNPs) were selected using the information available from the International HapMap Project. SNP selection was based on a randomly selected population with northern and western European ancestry by the Centre d'Etude du polymorphisme Humain (CEPH) [35]. The Minor Allele Frequency MAF had to be > 0.05 in order to exclude rare homozygous genotypes. Forty-two SNPs within the *CHRM2 *gene were thus selected from the CEPH population using Haploview version 3.32 (NCBI build 36.1).

SNP genotyping was performed using the SNPlex^® ^assay platform. The SNPlex assay was conducted following the manufacturer's recommendations (Applied Biosystems, Foster city, CA, USA). All pre-PCR steps were performed on a cooled block. Reactions were carried out in Gene Amp 9700 Thermocycler (Applied Biosystems, Foster city, CA, USA). Data was analyzed using Genemapper v3.7 (Applied Biosystems, Foster city, CA, USA).

### CHRM2 transcripts at brain level

Three different primer combinations were used to investigate the presence of CHRM2 transcript variants in normal brain controls. Forward primers F_A&B_GAGGCATCCAGGTCTCCAT, F_C&D_CGCAGCTCTCGCCA-GAGCCTT, and F_E&F_AAAGGACTCCTCGCTCCTTC were used in combination with a unique reverse primer R_A-F_CCCGATAATGGTCACCAAAC in order to tag isoforms A till F. PCR was performed at 94°C for 30 sec, 55°C for 30 sec, and 72°C for 1:30 min, for 40 cycles, followed by a 7 min extension at 72°C. To verify primers specificity PCR products were run on a 2% agarose gel.

### Gene expression assay

RT-PCR was performed using specific primers encompassing the untranslated exon 5 (the last untranslated exon), which is present in all mRNA transcripts, and the coding sequence (CDS) of the *CHRM2 *gene; F-GAAACCAGCGACAGGTTTAAATG, R-GCTATTGTTAGAGGA-GTTTGTTGAGTTATTC. PCR was carried out at 94°C for 1 min, 64°C for 1 min, and 72°C for 1 min, for 40 cycles, followed by a 10 min extension at 72°C. Optimization of primer concentration and cDNA input was performed and dissociation curves for the selected primers obtained. Two housekeeping genes – *β-actin *and *HPRT *– were used as internal controls. RT-PCR reactions were performed twice independently, each time in duplicate.

### Statistical analyses

Allele frequencies of all SNPs were estimated in both the children and adult cohorts using Haploview [36] in which a Hardy-Weinberg test is implemented, based on an exact calculation of the probability of observing a certain number of heterozygotes conditional on the number of copies of the minor SNP allele.

Genetic association tests were conducted using the program QTDT which implements the orthogonal model proposed by Abecasis *et al*., 2000 [20] (see also Fulker *et al*., 1999; Posthuma *et al*., 2004 [21,22]). This model allows one to decompose the genotypic effect into orthogonal between- (β_b_) and within- (β_w_) family components, and also models the residual sib-correlation as a function of polygenic or environmental factors. MZ twins can be included and are modelled as such, by adding zygosity status to the datafile. They are not informative to the within family component (unless they are paired with non-twin siblings), but are informative for the between family component. The between-family association component is sensitive to population admixture, whereas the within-family component is significant only in the presence of LD due to close linkage. The models used in QTDT take into account additive allelic between- and within family effects.

It is worth noting that, if population stratification acts to create a false association, the test for association using the within family component is still valid. More importantly, if population stratification acts to hide a genuine association, the test for association using the within family component has more power to detect this association than a population based association test. A significance level α of 0.01 was chosen.

## Results

**Figure 1:**
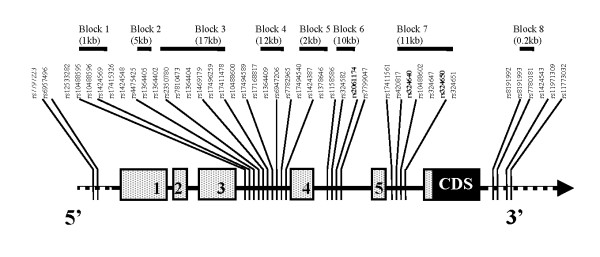
Location of single nucleotide polymorphisms (SNPs) within the *CHRM2 *gene on chromosome 7 and LD blocks defined by them, respectively. Coding sequence (CDS) is depicted in black. Untranslated exons (Exon 1 till Exon 5) are depicted in grey. SNPs already reported in our previous study (Gosso *et al*., 2006) are in **bold.**

Genotyping success rate was 95.36 (SD = 3.80) among both cohorts. Six *tag-*SNPs, (rs6957496, rs1424569, rs10488600, rs17494540, rs324582, and rs11773032), although with high genotyping rate, deviated from HWE (*P < 0.05*) despite a high genotype call rate. One *tag-*SNP, rs11773032 showed no variation in our population and was thus deleted from further analysis. LD parameters D' and r^2 ^were obtained for all successfully genotyped SNPs. LD blocks were generated applying the algorithm defined by Gabriel *et al*., 2002 [37] in which confidence bounds on D' are generated if 95% of the information shows "strong LD". By default, this method ignores markers with MAF < 0.05 (see Figure 1 and Table 2).

**Table 2:** SNPs descriptives for *young*, *adult *and *combined *cohorts

		rs#	Position^a^	*Tagged SNP*	LD_BLOCK_	MA	MAF Young	MAF Adult	HWE-pval	%Geno
1	*5'UTR*	rs7797223	136198443			T	0.25	0.26	0.77	95.6
2		**rs6957496**	136202377	1		G	0.09	0.11	0.02	96.6

3	*intron 3*	**rs12533282**	136207518	1, 4, 5,	1(1 kb)	G	0.18	0.17	0.40	98.6
4		rs10488595	136208970			A	0.18	0.17	0.71	97.7
5		rs10488596	136209134			T	0.18	0.16	0.37	97.2
6		**rs1424569**	136211219			A	0.44	0.47	0.02	94.8
7		**rs17415326**	136214872			C	0.02	0.05	0.48	95.1
8		rs1424548	136219956			T	0.37	0.36	0.53	98.2
9		**rs4475425**	136225739		2 (5 Kb)	A	0.21	0.24	0.87	94.8
10		**rs1364405**	136231025	41		A	0.35	0.33	0.08	97.9
11		**rs1364402**	136234903		3 (17 kb)	C	0.12	0.11	1.00	98.7
12		**rs2350780**	136243509			G	0.40	0.39	0.59	98.8
13		**rs7810473**	136246997			G	0.42	0.42	0.30	98.9
14		rs1364404	136248827			T	0.31	0.32	0.62	98.1
15		**rs1469179**	136251497	22		A	0.44	0.46	0.18	97.3
16		rs17496259	136251883			A	0.31	0.31	0.48	95.8
17		rs17411478	136251909			T	0.31	0.32	0.37	99.1
18		**rs10488600**	136255998			T	0.10	0.13	0.00	98.0
19		**rs17494589**	136256129	26		A	0.20	0.18	0.07	94.9
20		**rs17168817**	136258808			T	0.08	0.06	0.87	99.2
21		rs1364409	136262573		4 (12 kb)	T	0.32	0.35	0.13	96.4
22		rs6947206	136265651			C	0.46	0.48	0.12	94.0
23		**rs7782965**	136274673	21, 26, 27		T	0.32	0.35	0.45	90.4
24		rs17494540	136277380			C	0.20	0.18	0.01	96.3
25		rs1424387	136282543			C	0.31	0.31	0.39	99.0
26		rs1378646	136285541		5 (2 kb)	G	0.35	0.37	0.32	98.8

27	*intron 4*	rs1158586	136287676			G	0.34	0.40	0.42	93.0
28		**rs324582**	136301147			G	0.07	0.10	0.02	96.4
29		**rs2061174**	136311940	30	6 (10 kb)	G	0.34	0.35	0.93	84.6
30		rs7799047	136322098			G	0.34	0.35	1.00	93.5

31	*intron 5*	**rs17411561**	136332728	14, 16, 17, 25		C	0.32	0.25	0.25	87.7
32		rs420817	136337943		7 (11 kb)	C	0.48	0.47	0.21	95.7
33		**rs324640**	136339536	32		G	0.46	0.50	0.17	86.2
34		**rs10488602**	136341043			C	0.22	0.23	0.43	98.1
35		rs324647	136343292			C	0.14	0.15	0.13	95.9
36		**rs324650**	136344201			T	0.47	0.48	0.08	85.2
37		**rs324651**	136349801	35		T	0.14	0.14	0.13	93.1

38	*3'UTR*	**rs8191992**	136351848		8 (0.2 kb)	T	0.45	0.48	0.60	96.5
39		**rs8191993**	136352103			G	0.35	0.35	0.93	94.9
40		**rs7780181**	136357075			G	0.42	0.44	0.83	98.7
41		rs1424543	136360300			C	0.36	0.32	0.01	95.4
42		**rs11971309**	136362695	8		T	0.38	0.37	0.57	90.0
43		**rs11773032**	136391582			A	0.00	0.01	1.00	98.1

^a ^Chromosomal single nucleotide position (SNP) position based on *Build 36.1*. *Tag-*SNPs are depicted in **bold**. *Abbreviations*: LD, Linkage disequilibrium; MA, Minor Allele, MAF Minor Allele Frequency; HWE, Hardy-Weinberg Equilibrium

**Figure 2:**
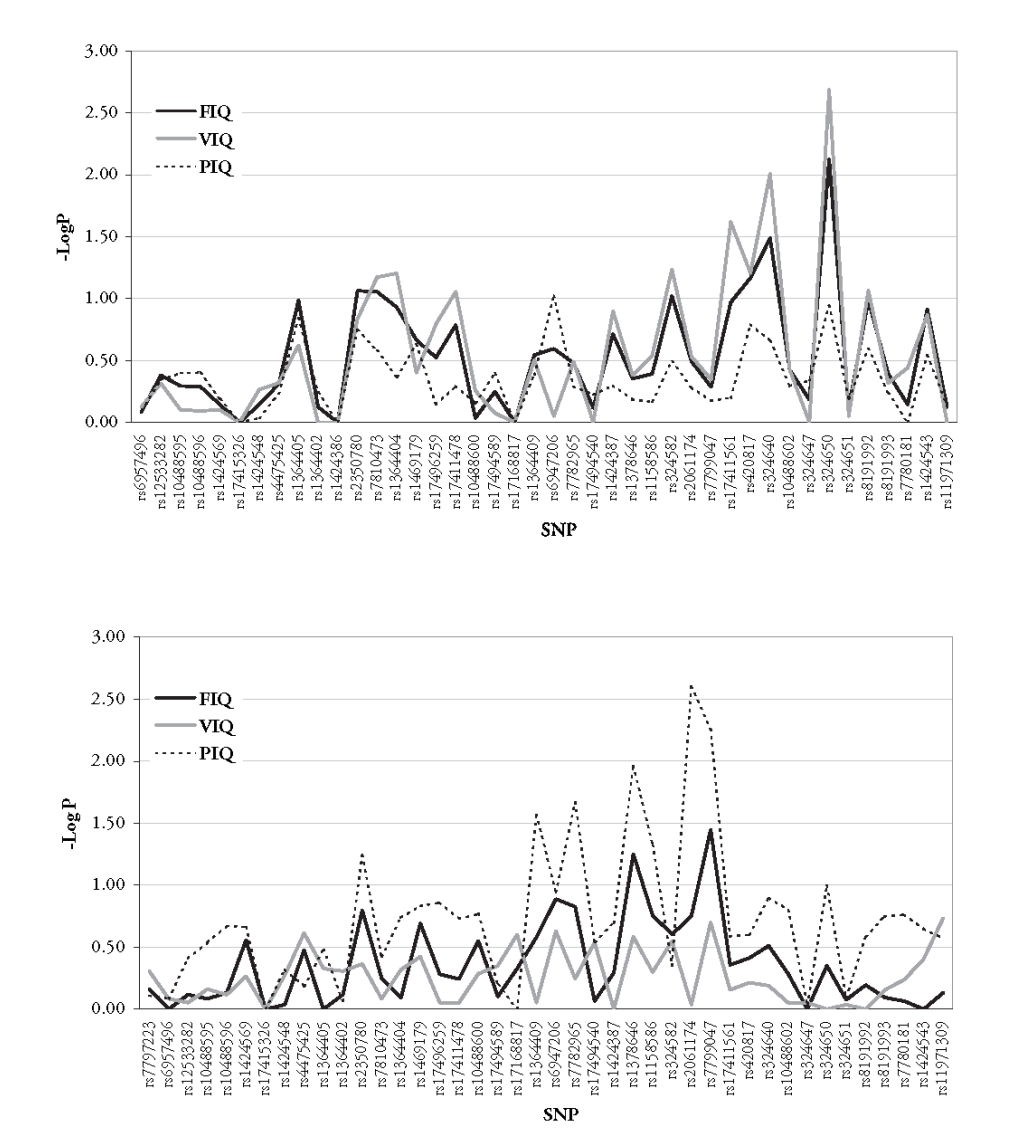
QTDT family-based results for *tag-SNPs *plotted against FSIQ, VIQ, and PIQ for *young *(A) and *adult *(B)cohorts.

Two 5'UTR SNPs, previously reported, showed the strongest association with IQ, rs2061174 (intron 4) in the *adult *cohort and rs324650 (intron 5) in the *young *cohort [8] (see Figure 2). Within-family genetic effects were reflected in an increased IQ of 6.89 (PIQ) points for those individuals carrying the "A" allele of rs2061174 within the *adult *cohort. individuals in the *young *cohort bearing the "T" allele of rs324650 showed an increment of 5.30 IQ (VIQ) points (see Tables 3, 4 and 5). Interestingly, the most significant variant in the *young *cohort, rs324650, is part of a short interspersed repeat (SINE), namely a MIRb (mammalian-wide interspersed repeat) repeat of 160 bp long. The derived "T" allele contained in this repeat seems to be human-specific. In addition this MIRb repeat is also present in non-human primate linages – rhesus (*macaca mulatta*) and chimpanzee (*pan troglodytes*) – but not in other mammalian linages. Such an allele-specific effect may reflect that the variant is in LD with the *causal *allele, or that the "T" allele is directly modifying binding-properties of transcription starting sites (TSS) [38].

**Table 3:** Means (SD) per genotype for PIQ, VIQ and FIQ for *young *and *adult *cohorts for the most significant SNPs within the *CHRM2 *gene

**SNP**		**Young Cohort**				**Adult Cohort**			
**position (bp)**	**Phenotype**	**Genotype Frequency**			**Total N**	**Genotype Frequency**			**Total N**
		**AA**	**AG**	**GG**		**AA**	**AG**	**GG**	
		*0.38*	*0.46*	*0.17*		*0.39*	*0.47*	*0.14*	
rs2350780	PIQ	94.43 (18.96)	95.21 (19.86)	95.94 (17.59)	366	104.77 (12.93)	104.61 (11.44)	104.37 (10.81)	359
(136243509)	VIQ	102.24 (13.67)	103.07 (12.69)	104.17 (11.67)	367	104.81 (13.56)	104.19 (11.00)	104.40 (11.43)	359
	FIQ	98.38 (15.54)	99.11 (15.26)	100.79 (13.70)	366	104.54 (12.82)	103.89 (10.50)	103.77 (10.18)	359
		**AA**	**AT**	**TT**		**AA**	**AT**	**TT**	
		*0.44*	*0.47*	*0.09*		*0.42*	*0.45*	*0.13*	
rs1364409	PIQ	95.30 (19.27)	93.93 (18.99)	97.16 (19.92)	361	105.16 (12.83)	104.52 (11.30)	104.31 (10.57)	350
(136262573)	VIQ	102.30 (13.91)	102.93 (12.01)	105.86 (12.48)	362	104.61 (12.56)	104.45 (11.76)	104.02 (10.85)	350
	FIQ	98.72 (16.00)	98.47 (14.31)	102.82 (15.57)	361	104.56 (12.20)	104.03 (11.03)	103.49 (9.34)	350
		**CC**	**CT**	**TT**		**CC**	**CT**	**TT**	
		*0.44*	*0.46*	*0.10*		*0.42*	*0.46*	*0.13*	
rs7782965	PIQ	95.14 (19.46)	93.97 (19.25)	96.66 (19.06)	345	104.35 (11.87)	104.81 (11.51)	104.50 (10.67)	345
(136274673)	VIQ	101.96 (14.03)	102.56 (11.75)	105.31 (13.17)	346	104.04 (12.35)	104.47 (11.61)	103.66 (10.93)	345
	FIQ	98.43 (16.17)	98.28 (14.35)	102.18 (15.52)	345	103.85 (11.53)	104.16 (11.08)	103.34 (9.35)	345
		**AA**	**AG**	**GG**		**AA**	**AG**	**GG**	
		*0.41*	*0.48*	*0.11*		*0,39*	*0,46*	*0,15*	
rs1378646	PIQ	95.87 (18.83)	93.78 (18.93)	96.80 (19.62)	365	104.52 (13.00)	104.92 (11.160	104.61 (10.59)	363
(136214872)	VIQ	102.21 (14.06)	103.03 (11.85)	104.41 (12.74)	366	104.06 (13.22)	105.03 (11.61)	103.87 (11.01)	363
	FIQ	98.97 (15.80)	98.48 (14.24)	101.62 (15.39)	365	103.98 (12.73)	104.55 (10.72)	103.50 (9.51)	363
		**AA**	**AG**	**GG**		**AA**	**AG**	**GG**	
		*0.44*	*0.44*	*0.12*		*0.42*	*0.47*	*0.11*	
rs2061174	PIQ	95.56 (18.61)	93.58 (20.13)	96.66 (18.12)	363	103.33 (12.81)	105.34 (11.33)	105.11 (9.40)	389
(136311940)	VIQ	101.55 (13.93)	102.89 (12.32)	106.34 (11.77)	364	103.60 (13.62)	105.36 (11.03)	102.64 (10.43)	389
	FIQ	98.40 (15.60)	98.40 (15.37)	102.14 (14.06)	363	103.16 (12.82)	104.93 (10.23)	102.88 (8.97)	389
		**TT**	**CT**	**CC**		**TT**	**CT**	**CC**	
		*0.48*	*0.42*	*0.10*		*0.59*	*0.34*	*0.07*	
rs17411561	PIQ	87.19 (19.37)	95.95 (18.21)	94.89 (19.64)	345	107.15 (11.31)	103.48 (11.47)	105.02 (11.83)	307
(136332728)	VIQ	99.53 (12.25)	103.45 (12.94)	103.71 (12.72)	346	108.09 (10.22)	103.21 (10.44)	104.54 (12.44)	307
	FIQ	92.72 (15.08)	99.94 (14.63)	99.54 (15.76)	345	107.24 (10.13)	102.78 (10.31)	104.39 (11.61)	307
		**AA**	**AG**	**GG**		**AA**	**AG**	**GG**	
		*0.29*	*0.50*	*0.21*		*0,25*	*0,49*	*0,26*	
rs324640	PIQ	94.72 (19.94)	94.04 (19.09)	96.61 (18.68)	363	102.21 (12.83)	105.64 (11.55)	104.37 (11.03)	386
(136339536)	VIQ	101.70 (13.90)	102.77 (12.79)	103.88 (12.38	364	102.81 (13.92)	105.69 (11.70)	103.35 (11.26)	386
	FIQ	97.88 (16.29)	98.53 (14.79)	100.83 (15.15)	363	102.08 (12.61)	105.36 (11.12)	103.17 (10.02)	386
		**AA**	**AT**	**TT**		**AA**	**AT**	**TT**	
		*0.30*	*0.48*	*0.21*		*0.26*	*0.48*	*0.26*	
rs324650	PIQ	93.59 (19.42)	94.45 (19.20)	96.82 (18.40)	363	102.59 (12.51)	105.50 (11.83)	104.19 (11.11)	369
(136344201)	VIQ	101.43 (13.98)	102.73 (12.76)	104.36 (11.92)	364	103.37 (13.52)	105.61 (11.69)	102.83 (11.48)	369
	FIQ	97.14 (15.99)	98.73 (14.95)	101.26 (14.60)	363	102.54 (12.28)	105.25 (11.38)	102.83 (10.21)	369

**Table 4:** Population and family-based QTDT results for *young *cohort for the most significant variants among *CHRM2 *gene

		Population-based				Family-based			
***position (bp)***	**Phenotype**	**N_POPULATION_**	**χ^2^**	**P**	**GE**	**N_FAMILY_**	**χ^2^**	**P**	**GE**
rs2350780	PIQ	366	0.74	0.390	1.34 (G)	95	1.81	0.179	3.63 (A)
(136243509)	VIQ	366	1.62	0.203	1.42 (G)	95	2.11	0.147	2.47 (A)
	FSIQ	366	1.82	0.177	1.68 (G)	95	2.94	0.086	3.48 (A)
rs1364409	PIQ	362	0.13	0.718	0.57 (T)	96	0.67	0.413	2.33 (A)
(136262573)	VIQ	362	1.46	0.227	1.42 (T)	96	1.02	0.313	1.84 (A)
	FSIQ	362	0.92	0.337	1.37 (T)	96	1.14	0.286	2.23 (A)
rs7782965	PIQ	346	0.17	0.680	0.77 (T)	85	0.18	0.671	2.00 (C)
(136274673)	VIQ	346	1.57	0.210	1.42 (T)	85	0.43	0.512	1.74 (C)
	FSIQ	346	1.03	0.310	1.37 (T)	85	0.94	0.332	2.05 (C)
rs1378646	PIQ	366	0.00	1.000	0.00 (G)	98	0.20	0.655	1.26 (A)
(136214872)	VIQ	366	0.88	0.348	1.03 (G)	98	0.66	0.417	1.39 (A)
	FSIQ	366	0.32	0.572	0.76 (G)	98	0.59	0.442	1.55 (A)
rs2061174	PIQ	363	0.01	0.920	0.19 (G)	111	0.41	0.522	1.69 (A)
(136311940)	VIQ	363	3.25	0.071	1.94 (G)	111	1.10	0.294	1.68 (A)
	FSIQ	363	1.10	0.294	1.37 (G)	111	0.98	0.322	1.91 (A)
rs17411561	PIQ	345	1.20	0.273	1.91 (C)	85	0.23	0.632	1.47 (C)
(136332728)	VIQ	345	2.51	0.113	1.81 (C)	85	5.09	0.024	4.35 (C)
	FSIQ	345	2.79	0.095	2.29 (C)	85	2.59	0.108	3.61 (C)
rs324640	PIQ	363	0.62	0.620	1.34 (G)	105	1.51	0.219	3.45 (A)
(136339536)	VIQ	363	2.83*	0.093	1.94 (G)	105	**6.67**	**0.010**	**4.59 (A)**
	FSIQ	363	2.39	0.122	1.98 (G)	105	4.57	0.033	4.42 (A)
rs324650	PIQ	363	1.65	0.199	2.10 (T)	100	2.51	0.113	4.40 (T)
(136344201)	VIQ	363	4.56*	0.033	1.42 (T)	100	**9.50**	**0.002**	**5.30 (T)**
	FSIQ	363	4.55	0.033	2.74 (T)	100	**7.14**	**0.008**	**5.35 (T)**

*Stratification significant at *P *= 0.05

*Note*: N denotes the number of individuals informative for the within family association test, i.e. those individuals that occur in families with more than one genotype. QTDT assumes equal genotypes for MZ twins and includes non-typed MZ co-twins with IQ scores. *Abbreviation*: GE genotypic effect (increaser allele).

**Table 5:** Population and family-based QTDT results for *adult *cohort for the most significant variants among *CHRM2 *gene

		Population-based				Family-based			
***Position (bp)***	**Phenotype**	**N_POPULATION_**	**χ^2^**	**P**	**GE**	**N_FAMILY_**	**χ^2^**	**P**	**GE**
rs2350780	PIQ	359	0.26	0.610	0.47 (A)	95	3.62	0.057	3.31 (A)
(136243509)	VIQ	359	0.01	0.920	0.12 (A)	95	0.62	0.431	1.26 (A)
	FSIQ	359	0.05	0.823	0.22 (A)	95	1.98	0.159	2.22 (A)
rs1364409	PIQ	350	0.15	0.699	0.35 (A)	92	4.90	0.027	3.13 (A)
(136262573)	VIQ	350	0.05	0.823	0.24 (A)	92	0.02	0.888	1.05 (A)
	FSIQ	350	0.05	0.823	0.22 (A)	92	1.25	0.264	0.72 (A)
rs7782965	PIQ	345	0.94	0.332	0.93 (C)	91	5.29	0.021	3.36 (C)
(136274673)	VIQ	345	0.24	0.624	0.49 (C)	91	0.33	0.566	0.16 (C)
	FSIQ	345	0.43	0.512	0.67 (C)	91	2.08	0.149	1.60 (C)
rs1378646	PIQ	363	1.08*	0.303	1.05 (A)	90	**6.48**	**0.011**	**3.77 (A)**
(136214872)	VIQ	363	0.61	0.435	0.73 (A)	90	1.27	0.26	1.10 (A)
	FSIQ	363	0.76	0.383	0.78 (A)	90	3.65	0.056	2.36 (A)
rs2061174	PIQ	389	4.64*	0.031	2.10 (A)	101	**9.14**	**0.003**	**6.89 (A)**
(136311940)	VIQ	389	0.06	0.806	0.24 (A)	101	0.01	0.920	1.78 (A)
	FSIQ	389	0.97	0.325	0.89 (A)	101	1.82	0.177	3.76 (A)
rs17411561	PIQ	306	0.15	0.699	0.47 (T)	79	1.28	0.589	0.69 (C)
(136332728)	VIQ	306	0.42	0.517	0.24 (T)	79	0.15	0.699	0.44 (T)
	FSIQ	306	0.02	0.888	0.11 (T)	79	0.60	0.439	0.08 (C)
rs324640	PIQ	386	2.37	0.124	1.40 (A)	123	2.36	0.126	3.05 (A)
(136339536)	VIQ	386	0.02	0.888	0.12 (A)	123	0.21	0.647	1.57 (A)
	FSIQ	386	0.54	0.462	0.67 (A)	123	1.04	0.308	2.22 (A)
rs324650	PIQ	369	2.09	0.148	1.28 (A)	117	2.69	0.101	1.69 (T)
(136344201)	VIQ	369	0.15	0.699	0.36 (A)	117	0.00	1.000	0.78 (T)
	FSIQ	369	0.13	0.718	0.33 (T)	117	0.58	0.446	0.77 (T)

*Stratification significant at *P *= 0.05

*Note*: N denotes the number of individuals informative for the within family association test, i.e. those individuals that occur in families with more than one genotype. QTDT assumes equal genotypes for MZ twins and includes non-typed MZ co-twins with IQ scores. *Abbreviation*: GE genotypic effect (increaser allele).

### CHRM2 transcripts expression at brain level and correlations with CHRM2 tag-SNPs

Previous studies have shown that of the six known isoforms of *CHRM2 *only C and D are expressed in the brain [17,18]. In contrast to this, we observed all six CHRM2 transcripts isoforms in brain material(data not shown).

After normalizing raw gene expression data to expression level of the housekeeping genes, no correlation between gene expression and *CHRM2 *gene genotypes for SNPs rs2061174, rs324640 or rs324650 was observed (data not shown).

## Discussion

Converging evidence from previous studies [7-9] has pointed to a role of the *CHRM2 *gene in intelligence. None of these studies, however, have identified the functional polymorphism explaining its role at a molecular level. The present study aimed to zoom in on the functional variants, by fine-mapping the most significant areas within this gene and also investigating differential brain expression as a function of different genotypes on the SNPs most strongly related to intelligence.

A total of 42 SNPs within the *CHRM2 *gene were genotyped in a *young *and *adult *cohort. Association analysis was conducted separately in both age cohorts to detect possible age dependent gene effects. Associations were found in different regions of the gene for each age cohort. Our current analyses showed that the same SNPs that were associated previously with intelligence, were again most significant, whereas a new SNP (rs2350780) showed a trend towards significance. Because of the dense coverage of SNPs used in this study, this confirms the importance of intron 4 and intron 5 regions, but rules out association with SNPs located elsewhere in the gene.

Four new SNPs in the intron 3 region, (rs2350780, rs1364409, rs7782965, and 1378646) showed association with PIQ in the *adult *cohort. These SNPs are in high LD (r^2 ^between 0.58 – 0.72) between the most significant SNPs. SNP rs2350780 and rs2061174 were also found to be associated with intelligence by Dick and co-workers [9]. These intronic SNPs are located 68 kb apart in introns 3 and 4, respectively. In our cohort, LD between these two variants is 0.58.

We found the most significant associations with PIQ in adults (rs2061174, χ^2 ^= 9.14; *P *= *0.003*) and with VIQ in children (rs323650, χ^2 ^= 9.50; *P *= *0.002*). Because only part of the variance in PIQ and VIQ is shared, and these results might reflect brain maturation processes and age-related genetic effects. Alternatively, the results could point to, and potentially explain, the genetic overlap between PIQ and VIQ, in which common genetic variants do not only interact modulating hippocampal neurotransmitter activity, but also and even more interesting from the epigenetic point of view, they might modulate priming and dendritic outgrowth underlying synaptic plasticity during embryogenesis [39] and at a post-natal stage [40], reflecting phenotypic variation at different IQ domains across the lifespan.

From a developmental perspective, brain maturation can be considered the most complex and dynamic lifelong process taking place in humans. Neuronal plasticity patterns (e.g. dendritic "pruning", synapse elimination, myelination) have been shown to vary significantly across life and among diverse brain structures (for a review see Toga et al., 2006 [41]). Variation in cognitive phenotypes may be the result of diverse allele-dependent effects that, although small in effect size, may contribute to cognitive phenotypes outcomes across life.

*In situ *hybridization experiments on mammals (e.g. mice) [42] have been of great utility to aid specific localization and interpretation of gene expression patterns. However, the localization of CHRM2 receptors transcripts has been conducted using probe sequences that did not distinguish between alternatively spliced transcripts. Our gene expression analyses showed that, in contrast to previously reported findings [17,18], all six currently known transcripts (isoforms A till F) of the *CHRM2 *gene were present in brain tissue.

Our genotype-dependent CHRM2 expression, did not reveal functional significance of any of the SNPs that were significantly related to intelligence. However, one should keep in mind that at this point we were only able to study material from superior and inferior parietal lobe and further studies on other brain regions might give different results. Furthermore it would be of interest to examine allele-specific effects on CHRM2 transcripts expression in much more detail, for example in relation to transcripts specific halve-life and their relation to LTP and memory.

Although brain expression analysis did not reveal differential expression of CHRM2 transcripts, our study further zooms in on the *CHRM2 *gene, clearly confirming two regions of most importance to intelligence within introns 4 and 5. These regions are poorly conserved regions among relatively distant species, although they are conserved among primate species. Interestingly, the variant associated in the *young *cohort (rs324650) is located within a SINE repeat (MIRb). SINE repeats belongs to a wide family of transposable elements, which constitute the largest class of interspersed repeats that are found in our genome (12%) together with long interspersed repeats (LINE) an long terminal repeats (LTRs) [43]. SINE repeats transpose through a RNA intermediate (reverse transcription process). All eukaryotic genomes contain mobile elements (retrosposable elements), although the proportion and activity of the classes of elements varies widely between genomes [44]. The *CHRM2 *gene, like its G-protein receptor counterparts, shares the interestingly feature – at least form a functional perspective – of being an *intronless *protein [45], which is also observed among dopamine receptors [46], widely studied in relation to attention deficits.

Recent research has revealed a potential functionality of retroposons in a gene-regulatory context [38,47-50]. It has been postulated that retroposon insertion processes may favour the generation of *intronless *proteins (for a review see Flavell 1995 and Brosius 2003 [51,52]). If this hypothesis holds, the resulting *intronless *proteins are expected to contain exons among their 5'UTR region. Not surprisingly, among G-proteins with intronless open reading frames (ORFs), about 18% have been reported to contain untranslated exons on their 5'UTR [46,53].

The majority of mammalian GPCRs are related to central nervous system activity, which often requires high and differential expression of many genes [53,54].

## Conclusion

Multiple promoters and transcripts have been reported for the *CHRM2 *gene suggesting that the associated regions we identified harbour functional elements involved in regulation of transcription and/or alternative splicing [17-19]. Further investigation involving functional assays and non-coding polymorphisms may aid the search and subsequent identification of regulatory variants underlying normal cognitive variation.

## Competing interests

The author(s) declare that they have no competing interests.

## Authors' contributions

MFG conducted the SNP selection and genotyping. MFG and DP performed the statistical analyses. DNA was provided by DIB. Phenotypic data was provided by DIB, EJC, TJC and DP. MFG drafted the manuscript under DP and PH supervision. DP and PH supervised the study. All authors read and approved the final manuscript.

## Pre-publication history

The pre-publication history for this paper can be accessed here:


